# Age Differences in Visual-Auditory Self-Motion Perception during a Simulated Driving Task

**DOI:** 10.3389/fpsyg.2016.00595

**Published:** 2016-04-28

**Authors:** Robert Ramkhalawansingh, Behrang Keshavarz, Bruce Haycock, Saba Shahab, Jennifer L. Campos

**Affiliations:** ^1^Research/iDAPT, Toronto Rehabilitation InstituteToronto, ON, Canada; ^2^Department of Psychology, University of TorontoToronto, ON, Canada; ^3^Institute of Medical Science, Faculty of Medicine, University of TorontoToronto, ON, Canada

**Keywords:** Multisensory Integration and Aging, self-motion perception, visual-auditory integration, principle of inverse effectiveness, older driver

## Abstract

Recent evidence suggests that visual-auditory cue integration may change as a function of age such that integration is heightened among older adults. Our goal was to determine whether these changes in multisensory integration are also observed in the context of self-motion perception under realistic task constraints. Thus, we developed a simulated driving paradigm in which we provided older and younger adults with visual motion cues (i.e., optic flow) and systematically manipulated the presence or absence of congruent auditory cues to self-motion (i.e., engine, tire, and wind sounds). Results demonstrated that the presence or absence of congruent auditory input had different effects on older and younger adults. Both age groups demonstrated a reduction in speed variability when auditory cues were present compared to when they were absent, but older adults demonstrated a proportionally greater reduction in speed variability under combined sensory conditions. These results are consistent with evidence indicating that multisensory integration is heightened in older adults. Importantly, this study is the first to provide evidence to suggest that age differences in multisensory integration may generalize from simple stimulus detection tasks to the integration of the more complex and dynamic visual and auditory cues that are experienced during self-motion.

## Introduction

The events that occur around us typically stimulate more than one sensory system simultaneously. It is well established that these congruent signals can promote better perceptual performance (i.e., faster and more reliable) than the constituent sensory signals presented in isolation (see Rowland and Stein, 2014; Stein et al., 2014 for a review). A growing body of evidence indicates, however, that this process may change with age (Laurienti et al., 2006; Peiffer et al., 2007; Mozolic et al., 2012). This is evidenced by the observation that the magnitude of the performance gains associated with congruent visual and auditory inputs is greater among older adults than it is among younger adults (e.g., Laurienti et al., 2006; Peiffer et al., 2007). Moreover, the magnitude of the performance decrements associated with conflicting visual and auditory inputs is greater among older adults than it is among younger adults (DeLoss et al., 2013; Guerreiro et al., 2013; Setti et al., 2013). That said, much of the current evidence to suggest that there are age differences in the integration of visual and auditory inputs is derived from stimulus detection and stimulus discrimination tasks (see Ernst and Bülthoff, 2004 for review). While precise and controlled, these tasks employ simple and highly discrete visual and auditory cues (e.g., flash of light, auditory beep; Shams et al., 2002) and thus, it is not clear whether this pattern of performance generalizes to other multisensory tasks. More recent research has revealed that the purported age differences in the interaction between visual and auditory cues is contingent upon the nature of the task (McGovern et al., 2014). For example, differences in multisensory enhancement have not been observed in tasks involving speech perception (Tye-Murray et al., 2010). The potentially stimulus and/or task dependent nature of the observation that there are age-differences in visual-auditory interactions is particularly important when considering their functional implications. Ultimately, previous studies have utilized sensory cues that are very different from the visual and auditory inputs that we typically encounter in our daily lives. For example, many of the tasks that we perform routinely involve moving through our environment and thereby elicit dynamic sensory inputs that must be combined continuously over time and space, not merely at discrete intervals (Campos and Bülthoff, 2012). In order to determine whether the purported age differences in the interaction between visual and auditory cues extend beyond the simple stimulus detection type tasks in which they have typically been observed, it is necessary to evaluate these sensory interactions during more dynamic, realistic tasks.

There is a great deal of evidence to demonstrate that there are age differences in visual, proprioceptive, and vestibular interactions during self-motion. Several previous studies have attempted to quantify the relative influence of individual sensory cues during locomotion in older and younger adults by manipulating the reliability or the nature of simultaneously presented visual and vestibular/proprioceptive cues. For example, Deshpande and Patla (2007) introduced perturbations of the vestibular system during goal-directed walking using galvanic vestibular stimulation. Younger adults appeared to be better able to down-weight the perturbed vestibular inputs than older adults, demonstrated by their superior ability to maintain a linear path toward their visual target. Further, Berard et al. (2012) reported that, when visual heading angles were dynamically changed while walking through a virtual environment, older adults were more greatly affected by this visual manipulation than were younger adults (both in terms of their final heading angle and dynamic walking parameters such as head/trunk/pelvis yaw angles). These findings suggest that there may be age-related changes in the way that dynamic sensory inputs interact during mobility-related tasks. However, very little previous work has investigated potential age differences specifically in the interaction between dynamic visual and auditory cues during self-motion. This is an important distinction because the mechanisms underlying visual-vestibular/proprioceptive cue interactions likely differ from those underlying visual-auditory cue interactions. Specifically, visual and vestibular cues generated during self-motion are idiothetic, as the observer’s own movements are the source of both the visual and the vestibular/proprioceptive cues that they receive. It has been posited that due to this inherent causal link between visual and vestibular/proprioceptive cues, they are integrated in a mandatory fashion (Prsa et al., 2012). Conversely, auditory cues are allothetic or generated by external sources and may thereby be integrated differently.

The important role that auditory cues play in self-motion perception has only recently been considered. It is well established that visual cues can provide a robust indication of self-motion with respect to, for instance, distance, and heading perception (Gibson, 1950; Warren and Hannon, 1988; Wilkie and Wann, 2002; Sun et al., 2004a,b; Frenz and Lappe, 2005; Fetsch et al., 2009; Butler et al., 2010; Campos et al., 2012). Optic flow alone can also be strong enough to elicit a strong illusory perception of actual self-motion in the absence of physical displacement (i.e., vection; Brandt et al., 1973). Growing evidence indicates that auditory cues can augment the visual perception of self-motion (Riecke et al., 2005, 2009; Keshavarz et al., 2014). For example, auditory cues can help an observer differentiate visual displacements caused by egomotion from those caused by the movement of external objects (Väljamäe et al., 2008; Calabro et al., 2011). Moreover, auditory cues are capable of strengthening the experience of vection (Riecke et al., 2009; Keshavarz et al., 2014). A representative example of a real-world task in which auditory cues demonstrably augment the visual perception of self-motion is driving a vehicle. When driving, we experience tire and wind turbulence noises that increase in amplitude relative to the rate at which we are traveling and therefore, these cues serve as a useful indication of speed (Merat and Jamson, 2011). The capacity for these cues to augment the visual perception of self-motion is illustrated by the fact that when no external feedback devices are available (i.e., speedometer) and auditory cues are removed, the perception of speed diminishes, causing drivers to underestimate their speed and/or to travel faster than intended (Horswill and Plooy, 2008). A number of investigations have also demonstrated that speed variability increases when driving without auditory cues (Matthews and Cousins, 1980; Horswill and Plooy, 2008; Merat and Jamson, 2011). Taken together, this evidence indicates that auditory cues bolster the perception of self-motion when presented in concert with visual motion cues. These observations also demonstrate that a visual-auditory driving task has the potential to help elucidate whether there are age differences in the interactions between visual and auditory cues that extend beyond simple stimulus detection tasks to more complex, continuous and dynamic sensory inputs under more realistic task conditions.

Therefore, in the current study we used a driving simulator to provide older adults with visual cues to self-motion (i.e., optic flow) while manipulating the presence or absence of congruent auditory inputs (i.e., engine, tire, and wind noise). The driving simulator allowed us to move toward more complex sensory inputs without relinquishing the experimental control afforded by more traditional visual-auditory stimulus detection tasks, as the simulator affords highly repeatable conditions and, unlike real-world driving, a simulation allows us to place tight constraints on task complexity, distraction, and other factors that may confound age differences in performance. Driving performance metrics (speed maintenance and lane keeping) then served as an assay of age differences in the interactions between visual and auditory cues. Based on previous driving research, we hypothesized that compared to driving with visual cues alone, speed accuracy would be improved and speed variability would be reduced with the addition of congruent auditory input (e.g., Denjean et al., 2012). But more importantly, based on previous evidence demonstrating age differences in the interaction between visual and auditory sensory inputs, we predicted that older adults would exhibit proportionally greater performance benefits than younger adults when congruent auditory and visual inputs were available compared to when only visual inputs were provided (e.g., Laurienti et al., 2006; Peiffer et al., 2007). While the auditory cues provided information about relative speed, they did not contain any information that was directly relevant to lane-keeping performance (e.g., no lane departure warning, rumble strips, etc.). Therefore, we predicted that lane-keeping performance would remain unchanged, unless auditory input were to promote more global changes in task performance by affecting, for instance, the participants’ general sustained attention, overall state of arousal, presence within the simulation or the perceived realism of the driving task (e.g., Cowan et al., 2015; Rojas et al., 2015).

## Materials and Methods

### Participants

Thirty-two older adults (65+ years) and twenty-three healthy younger adults (18–35 years) were recruited from the community. This study protocol was approved by the University Health Network research ethics board (REB 12-015-DE). All participants were prescreened to ensure that they held a valid driver’s license and had no serious medical conditions (e.g., seizures, stroke, heart condition), no physical conditions that may affect their driving ability (e.g., arm or leg injuries), did not use medications that may impair driving performance, and had no self-reported, uncorrected visual, or hearing impairments. All participants passed the Montreal Cognitive Assessment screening for mild cognitive impairment (≥ 26/30; Nasreddine et al., 2005). Participants were randomly assigned to one of two experimental groups: (1) visual cues alone or, (2) visual and auditory cues combined. Fourteen older adults (seven in the visual only condition, seven in the visual + auditory condition) and three younger adults in the visual only condition withdrew prior to completing the experiment due to symptoms of simulator sickness (for detailed discussion, see Keshavarz et al., 2015). The simulator sickness rates found here (50% older adults, 13% younger adults) are comparable to those that have been reported in previous driving simulator studies (e.g., Reed-Jones et al., 2008; Cassavaugh et al., 2011; Stoner et al., 2011). Due to simulator malfunction, data was not recorded for one younger adult and for two older adults in the visual + auditory condition. All 17 cases of incomplete data due to simulator sickness and technical issues were excluded from data analyses. **Table 1** summarizes the characteristics of the participants who had complete data and who were included in our analyses. Note that the high attrition rate led to a difference in the mean age between the older adults that comprised the visual only sensory condition and the older adults that comprised the visual auditory condition, but it is not expected to be confounded with the effects of sensory condition as will be discussed below.

**Table 1:** Participant demographics by age and sensory condition.

	Visual only				Visual + auditory			
	Younger		Older		Younger		Older	
	*N*	*M age* (*SD*)	*N*	*M age* (*SD*)	*N*	*M age* (*SD*)	*N*	*M age* (*SD*)
Male	4	27.25 (1.70)	6	76 (6.92)	5	26.2 (3.03)	6	65.67 (3.20)
Female	6	25.67 (5.27)	2	70 (4.24)	5	26.6 (2.61)	2	70 (5.66)
Overall	10	26.30 (4.13)	8	74.50 (6.67)	10	26.4 (2.67)	8	66.75 (3.99)

### Design

There were two between-subjects variables: age group (younger vs. older) and sensory condition (visual only vs. visual + auditory). Additionally, there were two within-subjects variables: drive number (acclimatization, 2, 3, 4, 5) and road geometry (straight vs. curved road segments). The result was a 2 (age) × 2 (sensory condition) × 5 (drive number) × 2 (road geometry) mixed factorial design.

### Apparatus and Stimuli

**FIGURE 1:**
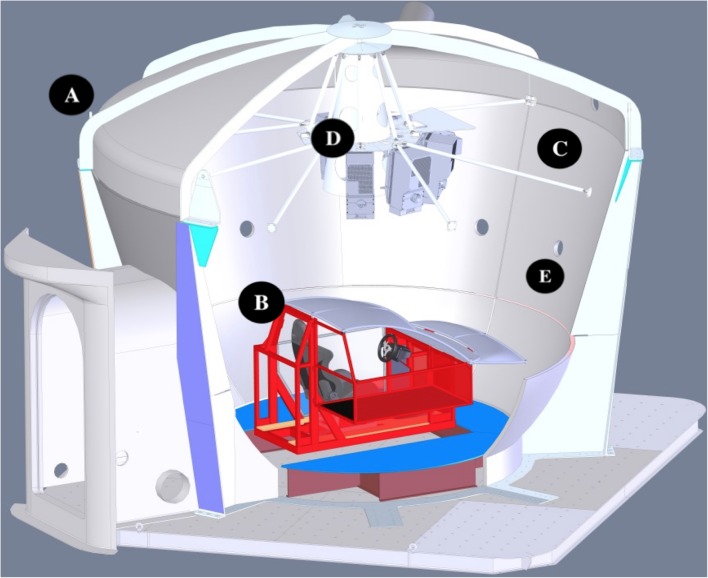
**Solidworks^TM^ rendered cutaway view of StreetLab, a fiberglass dome that can be configured into different virtual reality environments (A).** The driving task configuration consisted of a mock cab comprised of a steel frame, plastic body panels, a real car seat, a Logitech steering wheel and pedals, and a digital speedometer **(B)**. StreetLab contains a curved projection screen **(C)**, and six Eyevis ESP-LED projectors **(D)** that generate a 240° horizontal × 105° vertical field of view image. Vehicle and road contact sounds were conveyed over a 7.1 channel sound system. The center channel speaker is depicted **(E)**.

**FIGURE 2:**
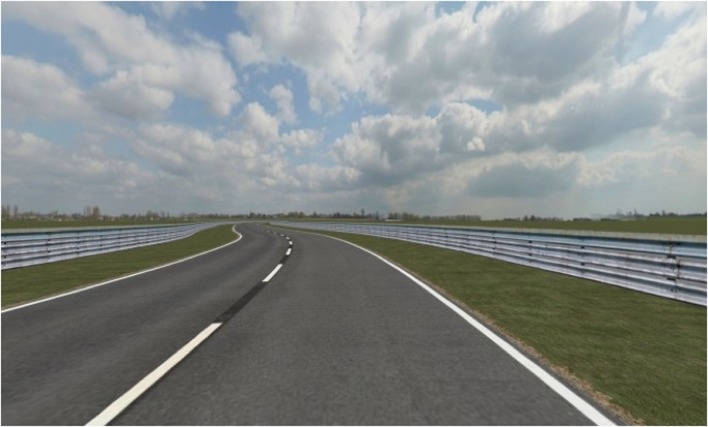
**Screenshot of the driving scene consisting of a two lane roadway with guardrails on either side and agricultural scenery on the horizon**.

The driving task took place within StreetLab, an immersive Virtual Reality laboratory housed within the Challenging Environment Assessment Laboratory at the Toronto Rehabilitation Institute’s iDAPT Centre for Rehabilitation Research (see **Figure 1A**). StreetLab was outfitted with a basic driving interface, consisting of a half-cab structure the approximate dimensions of a Smart car, which contained a car seat, a Logitech steering wheel and gas/brake pedals, and a digital speedometer (see **Figure 1B**). The dome-shaped lab contained an immersive, curved projection screen (see **Figure 1C**). The visual driving scene was rendered using the irrLicht engine and presented using six synchronized projectors (Eyevis ESP-LED; **Figure 1D**) each with a resolution of 1920 × 1200 for a total field-of-view of 240° horizontally and 105° vertically at 6.5 arcmin/OLP. The imagery was updated at 60 Hz, with a total time delay of approximately 50 ms between the driver inputs and the visual display of the outside world. The visual driving scene consisted of clear daytime driving conditions on a two lane rural road with guardrails and a series of left and right curves as shown in **Figure 2**. There were an equal number of left and right hand curves, with one of three radii: 400 m, 800 m, or 1200 m. The roadway was surrounded by an open grassy area with agricultural scenery (i.e., farms with barns and silos on the far horizon) that mainly provided optic flow information. No other moving objects (e.g., vehicles, pedestrians, animals, etc.) or obstacles were in the scene. Our goal was to capture a substantial duration of driving performance (≥ 25 min) to ensure that drivers had sufficient time to acclimatize to the simulator. This was an important consideration because older adults take a longer amount of time to acclimatize to driving simulators than younger adults do (e.g., Kawano et al., 2012). But to mitigate the risk of inattention, fatigue, and simulator sickness associated with driving for extended periods of time without interruption (e.g., Fowlkes et al., 1987; Philip et al., 2003; Yanko and Spalek, 2013), we created five separate courses that each took 5–7 min to complete. Each course was comprised of the same straight and curved road segments and thus each drive was identical in terms of their complexity and difficulty but the segments were arranged in different sequences so that drivers could not learn the courses. In addition to thwarting adverse effects, this approach allowed us to observe how participants’ performance changed as they progressed through each subsequent drive and to discern whether the rate at which participants adapted to the driving simulator was affected by age and/or the available sensory inputs.

The vehicle dynamics were developed in the MathWorks’ Simulink environment and were then compiled and run in real-time using Quanser’s QUARC operating system. The auditory stimuli were created by the IrrKlang sound engine (Gebhardt, 2009) and consisted of looped, digital recordings of (i) the engine from a 2007 Volkswagen Passat diesel, (ii) tire-road contact sounds, and (iii) brown noise to represent air rushing over the vehicle (Freesound.org, 2010). The frequency of the engine sounds scaled according to the speed of the vehicle in a linear fashion. The amplitude of the road contact and wind sounds scaled according to speed in an exponential manner. Sounds were presented to the driver using a 7.1 channel sound system. The system consisted of seven, 4′′ inch satellite loudspeakers (Meyer Sound MM-4XP) located behind the sound-permeable surface of the projection screen and a 10′′ subwoofer (Meyer Sound MM-10XP) located on the floor of the lab. The center channel speaker was positioned near head height at 0° azimuth (see **Figure 1E**) and the subwoofer was positioned below it. The other six loudspeakers were distributed in an array along the same horizontal plane as the center channel speaker at ± 28°azimuth (right front, left front), ± 90° azimuth (right side and left side), and ± 127.5° azimuth (right rear and left rear). Each speaker was positioned at a distance of 2.14 m from the participant. At 80 km/h, sound pressure level was 90 decibels (A-weighting). For each of the five drives, performance was measured by capturing speed (km/h), standard deviation in speed, and root mean squared error (RMSE) of lateral position (m) at a rate of 200 Hz over the course of the five drives. Performance measures were also separated by road geometry (i.e., straight vs. curved road segments), given that traversing curved road segments represented a higher level of task complexity.

### Self-report Measures

Participants were asked to rate the realism of the major components of the simulation including the steering, gas, brakes, accelerator, and the overall driving feel using a series of 7-point Likert scales. We also asked participants to report on the strength of their experience of vection (i.e., the sensation of self-motion in the absence of physical movement; see Hettinger et al., 2014) on a scale from 0 (none) to 10 (very strong). Additional control measures, including the Motion Sickness Susceptibility Questionnaire (MSSQ; Golding, 2006) were employed to measure proneness to motion sickness. The Fast Motion Sickness scale (FMS, (Keshavarz and Hecht, 2011) was used to track the severity of simulator sickness on a scale from 0 (no nausea) to 20 (severe nausea) during the simulation. After the driving task, the well-established Simulator Sickness Questionnaire (Kennedy et al., 1993) was administered to capture different symptoms of simulator sickness after completing the driving task. These measures were used to account for changes in sickness that may have coincided with age and/or with the available sensory input (see Keshavarz et al., 2015 for a full summary of the simulator sickness results).

### Procedure

Once informed consent was obtained, the medical and driving history questionnaires were administered, along with the MSSQ and the MoCA. Participants were then randomly assigned to either the visual only or the visual + auditory condition of the driving task (between-subjects). Participants were seated inside the driving simulator where they were instructed to maintain a target speed of 80 km/h, to adhere to the center of their lane, and to drive the simulator as they would their own vehicle. The participant then embarked on each of the five road courses. The order in which the courses were presented was counterbalanced. The first course that participants received always served as an acclimatization period that allowed them to become familiar with the feel of the simulator’s controls. During this acclimatization period, a digital speedometer was present to assist participants in achieving and maintaining their target speed of 80 km/h. The four subsequent drives served as experimental drives in which the speedometer was occluded and participants were to rely only upon the available sensory inputs in order to estimate and maintain their 80 km/h target speed.

At the start of each drive, the vehicle was stationary and the participant was instructed to bring the vehicle up to 80 km/h and to maintain this speed to the best of their ability. They were instructed to then decelerate slowly and to bring the vehicle to halt at the end of the drive, which was demarcated by the discontinuation of the paved roadway and guardrails. These acceleration and deceleration periods were excluded from the data analyses. To limit the possibility of participants forgetting the 80 km/h target speed due to memory decay and to constrain the experience of speed adaptation, a phenomenon whereby the visual perception of speed diminishes with prolonged exposure (Evans, 1991), we included a refresher drive between each experimental drive. During the refresher drives, the speedometer was made visible again and the driver was instructed to accelerate to 80 km/h and to maintain 80 km/h for a period of 60 s. Once this period was complete, they were instructed to return to a complete stop and the subsequent experimental drive was initiated. Data from these refresher periods were also excluded from the analyses.

For the duration of the simulation, the researcher sat inside the lab and asked the participant to report their level of sickness on the FMS scale once every 60 s over the duration of the five experimental drives and the interleaving refresher sessions. At the end of each experimental drive, participants were offered a break from the simulation, which nearly all participants declined. Once all experimental drives were complete, participants were asked to rate the realism of the components of the simulation along with the strength of the vection that they experienced.

## Results

Our primary objective was to examine driving performance across the experimental drives during which the speedometer was occluded and participants were required to rely only on the available sensory information (drives 2–5). Thus, we employed a series of mixed factorial ANOVAs with the between-subjects factors age (younger vs. older) and sensory condition (visual only vs. visual + auditory) and the within-subjects factors road geometry (straights vs. curves) and drive number (2, 3, 4, 5). A priori alpha level was set to α = 0.05. The Bonferroni correction for multiple comparisons was applied to all *post hoc* tests.

### Mean Speed

**FIGURE 3:**
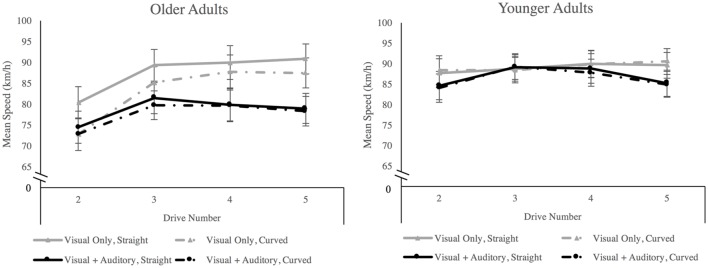
**Mean speed separated by age group, sensory condition, road geometry, and drive number.** Error bars are ±1SE. There were significant main effects of age group and of road geometry. Participants drove faster in drives 3, 4, and 5 than in drive 2 but drove slower on curved segments than on straight segments overall. There was an age × sensory condition × road geometry interaction in which older adults in the visual only condition traversed curved road segments at a lower rate than straight road segments.

Mauchly’s test of sphericity indicated that the assumption of sphericity had been violated for the factor drive number, χ^2^(5) = 11.38, *p* = 0.026, therefore degrees of freedom were corrected using Greenhouse–Geisser estimates of sphericity (ε = 0.78). There was a main effect of age group, *F*(1,32) = 4.35, *p* = 0.045, $\eta_{p}^{2}$ = 0.12, in which older adults drove more slowly (*M* = 81.81, *SE* = 2.19) than younger adults (*M* = 87.94, *SE* = 1.96). We observed a main effect of road geometry, *F*(1,32) = 17.39, *p* < 0.001, $\eta_{p}^{2}$ = 0.35, in which drivers drove more slowly on curved road segments (*M* = 84.19, *SE* = 1.44) than on straight road segments (*M* = 85.56, *SE* = 1.51). We also observed a main effect of drive number, *F*(2.37,76.09) = 6.99, *p* < 0.001, $\eta_{p}^{2}$ = 0.18. A *post hoc* Bonferroni test revealed that participants drove at higher speeds in the third, fourth, and fifth drives than in the second drive (see **Figure 3**). There was a significant three-way Age Group × Geometry × Sensory Condition interaction, *F*(1,32) = 9.38, *p* = 0.004, $\eta_{p}^{2}$ = 0.23. Older adults drove significantly slower when traversing curved road segments compared to straight road segments in the visual only sensory condition, but not in the visual + auditory condition (see **Figure 3**). There was also a significant Age Group × Geometry interaction *F*(1,32) = 15.529, *p* < 0.001, $\eta_{p}^{2}$ = 0.33 in which older adults traversed curved road segments at a lower rate (*M* = 80.47, *SE* = 2.16) than they traversed straight road segments (*M* = 83.15, *SE* = 2.25), and at a lower rate than younger adults traversed curved road segments (*M* = 87.91, *SE* = 1.93). No other effects or interactions were significant (*F* ≤ 3.73, *p* ≥ 0.062).

To more closely examine the impact of the available sensory inputs on speed accuracy, one-sample t-tests were used to compare mean speed in each sensory condition against the target value of 80 km/h for each age group and road geometry, aggregating performance across the experimental drives. For younger adults, speed was significantly greater than the target in all comparisons (*t* ≥ 3.49, *p* ≤ 0.007). For older adults, speed was only significantly greater than the 80 km/h target when traversing the straight road segments in the visual only condition *t*(7) = 2.72, *p* = 0.030). Otherwise, older adults’ speed did not differ significantly from the 80 km/h target.

### Standard Deviation in Speed

**FIGURE 4:**
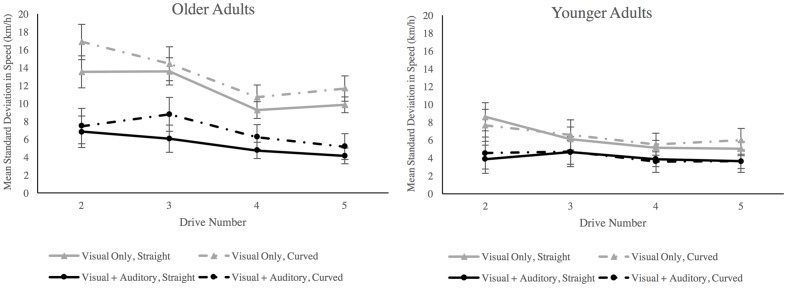
**Standard deviation in speed separated by age group, sensory condition, road geometry and drive number.** Error bars are ±1SE. There was an age × sensory condition interaction in which older adults exhibited less variability in speed when both visual and auditory inputs were available compared to when only visual inputs were available. Note also main effects of age group and of sensory condition.

Mauchly’s test of sphericity indicated that the assumption of sphericity had been violated for the factor drive number, χ^2^(5) = 38.85, *p* < 0.001, therefore degrees of freedom were corrected using Greenhouse–Geisser estimates of sphericity (ε = 0.56). All main effects were significant. There was a main effect of age group, *F*(1,32) = 19.00, *p* < 0.001, $\eta_{p}^{2}$ = 0.37, in which older adults exhibited a higher standard deviation in speed (*M* = 9.33, *SE* = 0.70) than younger adults (*M* = 5.20, *SE* = 0.63). There was a main effect of sensory condition in which standard deviation in speed was lower in the visual + auditory condition (*M = 5*.12, *SE* = 0.67) than in the visual only condition (*M* = 9.41, *SE* = 0.670). There was a main effect of road geometry, *F*(1,32) = 6.24, *p* = 0.018, $\eta_{p}^{2}$ = 0.16, in which standard deviation in speed was greater on curved road segments (*M* = 7.72, *SE* = 0.602) than on straight road segments(*M* = 6.81, *SE* = 0.39). There was a main effect of drive number, *F*(3,96) = 6.60, *p* < 0.001, $\eta_{p}^{2}$ = 0.17. A *post hoc* Bonferroni test (α = 0.05) revealed that the standard deviation in speed was lower in the fourth (*M* = 6.13, *SE* = 0.501), and fifth (*M* = 6.14, *SE* = 0.528) drives than in the second drive (*M* = 8.67, *SE* = 0.76), see **Figure 4**. We also observed a significant Age Group × Sensory Condition interaction, *F*(1,32) = 4.49, *p* = 0.042, $\eta_{p}^{2}$ = 0.123. For older adults, the addition of auditory inputs yielded a lower standard deviation in speed than visual input alone. For younger adults, there was no difference in performance between sensory conditions (see **Figure 4**). No other interactions were significant (*F* ≥ 4.08, *p* ≥ 0.052). Older adults in the visual only condition maintained a significantly greater mean speed than those in the visual + auditory condition. This greater speed could have inflated speed variability, thereby confounding the effect of sensory condition on speed variability. Thus, we transformed *SD* speed into *z*-scores to normalize *SD* speed across groups and submitted them to the 4-way ANOVA to confirm our observations. The Age Group × Sensory Condition interaction remained significant, *F*(1,32) = 5.19, *p* = 0.029, $\eta_{p}^{2}$ = 0.140, and *post hoc* Bonferroni tests confirmed that older adults in the visual only condition (*M* = 1.08, *SD* = 1.9) were significantly more variable in the speed that they maintained than those in the visual +auditory condition (*M* = -0.19, *SE* = 0.19).

### Lateral Control

RMSE lateral position was examined for the experimental drives, comparing between age groups, sensory conditions, and road geometry. No effects were significant for this parameter. There were no significant main effects of age group, *F*(1,32) = 3.00, *p* = 0.09, $\eta_{p}^{2}$ = 0.09; sensory condition, *F*(1,32) = 2.87, *p* = 0.09, $\eta_{p}^{2}$ = 0.08; road geometry, *F*(1,32) = 2.76, *p* = 0.11, $\eta_{p}^{2}$ = 0.08; or drive number, *F*(1,32) = 1.85, *p* = 0.14, $\eta_{p}^{2}$ = 0.05. No interactions were significant.

### Baseline Performance across Groups

Older adults in the visual only condition were significantly older (*M* = 74.5, *SE* = 2.36) than older adults in the visual + auditory condition (*M* = 66.75, *SE* = 1.41), *t*(14) = 2.82, *p* = 0.014. The mean age of the visual only older adult group was inflated by three older adults who were above the age of 75. To ensure that differences in performance were due to the available sensory inputs and not due to baseline differences in performance related to the disparities in the demographic composition of each group within our design, we first examined driving performance across groups within the acclimatization drive. In this drive, all participants were able to view the speedometer and thus any differences in the capacity to maintain the target speed would ostensibly stem from participants’ inherent performance variability. Ultimately, there were no differences across all groups in mean speed, standard deviation in speed, or RMS lateral position within the acclimatization period (*t* ≤ 1.15, min *p* ≥ 0.268). Further, we also used bivariate correlations to examine the relationship between age and each of the driving performance measures aggregated across the four experimental drives. There were again no significant correlations observed for any of the driving parameter measures across all of the groups, apart from one significant positive correlation between age and speed variability for the older adult visual only group (*r* = 0.83, *p* = 0.010), which we address in the Discussion.

### Perceived Realism

To examine how the experience of realism changed across the available sensory conditions, we analyzed each of the self-report measures pertaining to realism using a series of two-way, Age × Sensory Condition ANOVAs. Non-parametric analyses were also performed to confirm the veracity of our ANOVA results. For “overall driving feeling” there was no effect of age group, *F*(1,30) = 2.33, *p* = 0.14, $\eta_{p}^{2}$ = 0.07, but there was a main effect of sensory condition, *F*(1,30) = 10.51, *p* = 0.003, $\eta_{p}^{2}$ = 0.26, in which the visual + auditory condition was rated as being significantly more realistic (*M* = 5.2, *SE* = 0.36) than the visual only condition (*M* = 3.5, *SE* = 0.38). This observation was confirmed with a Mann–Whitney *U* test, (*U* = 60.50, *p* = 0.003 two-tailed). For the realism of the steering, braking, gas pedal, and the strength of vection, no effects were significant.

### Simulator Sickness

To assess the relationship between simulator sickness and driving performance, we took the total SSQ score and the peak FMS score of the participants who completed the experiment and correlated those scores with each driving performance measure, aggregated across the experimental drives and across road geometry. There were only two significant bivariate correlations: for younger adults in the visual only condition, peak FMS score was negatively correlated with standard deviation in speed (*r* = -0.83, *p* = 0.003) and for older adults in the visual auditory condition, total SSQ score was negatively correlated with mean speed (*r* = -0.77, *p* = 0.035). Ultimately, the current evidence does not suggest that increased simulator sickness led to diminished driving performance or that poor driving performance exacerbated simulator sickness.

## Discussion

There is growing evidence to suggest that the manner in which visual and auditory sensory inputs are integrated may change in late adulthood, but most of this evidence is derived from simple stimulus detection or stimulus discrimination tasks (e.g., Laurienti et al., 2006). These tasks are powerful and highly controlled, but much remains to be understood about how these effects generalize to other types of tasks and/or whether there are functional consequences associated with age-related changes in multisensory integration. Thus, we developed a multisensory driving task in which we systematically manipulated the presence or absence of congruent auditory input and used subsequent driving performance to index age differences in the interaction between visual and auditory cues. We predicted that auditory input would affect driving performance measures associated with speed (but not with lane keeping) and that these effects would be proportionally greater among older adults. When we examined standard deviation in speed during the experimental drives, we observed that speed variability was lower in the visual + auditory condition relative to the visual only condition for both younger and older adults. This pattern of observations aligns well with previous driving research indicating that compared to driving with visual input alone, speed variability is reduced in the presence of congruent auditory input (e.g., Denjean et al., 2012). But more importantly, the magnitude of these benefits was greater among older adults compared to younger adults. This observation is generally consistent with the findings reported by basic psychophysical studies exploring age-related changes in multisensory integration. Specifically, they mirror the observation that congruent visual and auditory cues confer greater gains in performance for older adults than for younger adults, compared to the constituent unisensory inputs presented in isolation of one another (e.g., Laurienti et al., 2006; Peiffer et al., 2007).

When we examined mean speed, we observed that older adults drove at significantly slower speeds than younger adults, ultimately traveling under the target speed of 80 km/h at the outset of the driving task. Older drivers have a tendency to self-regulate their behavior in order to minimize crash risk (see Charlton et al., 2003). This can include reducing speed when faced with challenging scenarios (e.g., Trick et al., 2010). In the current investigation, older adults may have traveled at a lower rate of speed compared to younger adults in order to maintain broad safety margins as they acclimatized to the driving task, a process that can take longer for older adults to complete (e.g., Kawano et al., 2012). Conversely, younger adults are known to drive faster than older adults in both real vehicles and in driving simulators (see Mullen et al., 2011 for review) and do so regardless of the prevailing task demands (e.g., Trick et al., 2010). Therefore, the overall differences in speed between older and younger adults were generally consistent with previous driving research examining age differences in performance. But more importantly, we also observed evidence to suggest that older adults were more greatly affected by the presence of combined visual and auditory inputs when estimating and maintaining their speed. In the visual only condition, older adults significantly reduced their speed in order to traverse the curved road segments but in the visual + auditory condition, older adults maintained nearly identical speeds on the straight and curved road segments. The presence of auditory cues allowed older adults to maintain speeds that were lower on average and ultimately closer to their 80 km/h target speed. This speed may have been more suitable for negotiating both straight and curved road segments and thus no changes in speed were required. In the visual only condition, the absence of auditory cues may have left older adults with a diminished capacity to estimate their speed, leading them to drive faster than intended (e.g., Evans, 1970; Horswill and Plooy, 2008) and thereby requiring them to reduce their speed in order to retain control over their vehicle when negotiating curved road segments. That is not to say that auditory cues provided an absolute measure of speed, but rather that the combination of visual and auditory input augmented older adults’ perception of relative speed. The interaction between age and sensory condition that we observed, particularly in the dimension of speed variability, suggests that age related changes in the interaction between visual and auditory cues as observed in the context of simple stimulus detection and discrimination tasks may extend to the continuous and dynamic visual and auditory cues that we encounter in our daily lives. This also suggests that age-related changes in the interaction between visual and auditory cues may have important implications for the way that older adults perform everyday multisensory tasks including, but not limited to, driving a motor vehicle.

That said, there are a number of additional factors that may have contributed to the pattern of observed performance that must be addressed. For instance, the presence of auditory cues may have had a broad influence on task performance by modulating more global factors, such as enhanced sustained attention, increased state of arousal, greater sense of presence in the simulation, or a greater sense of perceived realism. However, we did not observe evidence of global changes across all aspects of driving task performance, rather, only the driving parameters that we predicted would be affected by auditory feedback (i.e., speed perception) were influenced by the availability of auditory cues. Lane keeping, a driving parameter that we predicted would *not* be affected by auditory feedback was not influenced by the availability of auditory cues. The specificity of these performance outcomes indicates that auditory cues influenced driving performance by augmenting speed perception rather than by exerting a global influence on task performance. However, an important factor that may have contributed to age-related differences in performance was cumulative driving experience. While the age differences in performance that we observed may stem from age differences in multisensory self-motion perception, they may also reflect age differences in years/km lifetime driving experience. As driving experience accumulates, a number of important cognitive and perceptual changes occur as a function of this experience. For instance, drivers who have traveled between 10,000 and 50,000 km begin to develop the ability to rely on the ambient or peripheral visual channel to govern lateral position (Summala et al., 1996; Horrey et al., 2006). It is also possible that in parallel, drivers learn with increasing experience how the frequency and amplitude of engine and road/tire noises scale according to speed and learn to use this information help govern speed (e.g., Merat and Jamson, 2011). This learned reliance on auditory information for speed perception may lead older adults to be more susceptible to changes in performance due to the presence or absence of auditory cues. Thus, future research in this domain should seek to employ multisensory tasks in which the relative effects of age and previous experience can be parsed.

### Limitations

Our high attrition rate coupled with our between subjects design made it difficult to maintain groups that were well matched in terms of age and gender. For example, there was a difference in the mean age of the older adults in the visual only condition and the older adults in the visual + auditory condition. This is an important consideration because performance becomes increasingly variable with advanced age across several domains (e.g., Hultsch et al., 2002). Indeed, we observed a positive correlation between age and speed variability within the visual only older adult group. However, age differences alone cannot account for the effect of sensory condition, given that the two groups were no different in their driving performance at baseline. Gender differences in driving performance are also an important factor to consider but the modest number of men and women and relative imbalance within each cell of our design does not permit us to make meaningful comparisons between men and women. Future investigations should consider gender differences in unisensory and multisensory driving performance.

An additional limitation associated with our sample was that we utilized self-report measures to screen for sensory impairment, which can be unreliable. While we assume that the older adults in our sample were within the normal hearing range, it is possible that a clinical audiometric examination would reveal some degree of age-related hearing loss (see Pichora-Fuller and MacDonald, 2009). Hearing loss is an important factor to account for in the context of driving performance, given associations have been shown between the risk of having a collision and hearing loss (e.g., Picard, 2008; Hickson et al., 2010). Future studies should incorporate central and peripheral audiometric testing to better understand the association between hearing status and driving performance during multisensory driving tasks.

Finally, our driving task was not able to precisely quantify the relative contributions of visual and auditory inputs to this task or to determine whether they were optimally integrated. In order to achieve this, one would have to obtain performance measures during both unimodal (vision alone and auditory alone) and bimodal conditions. Because it is impossible to control a motor vehicle with auditory cues alone, we were only able to examine how combining visual cues with congruent auditory cues affected driving performance and age differences therein.

## Conclusion and Future Directions

The goal of the present study was to determine whether evidence of age differences in visual-auditory cue integration would be observed in the context of real-world multisensory tasks that involve continuous and dynamic sensory inputs. We found that both younger adults and older adults exhibited a reduction in speed variability in the presence of congruent visual and auditory cues compared to visual cues alone, but this effect was greater among older adults. This finding provides preliminary evidence to suggest that age differences in multisensory integration may generalize to more complex sensory inputs and that heightened multisensory integration may carry functional implications for older adults in the context of self-motion and mobility-related tasks. Our observations could also have important implications for the design of real vehicles. For example, automakers are moving toward quieter interiors by utilizing advanced sound deadening materials (Hellier et al., 2011) and even by employing active noise cancelation technologies (Hansen and Snyder, 1996; Hansen, 2002; Wang and Wang, 2012). But these initiatives could be inadvertently removing information that otherwise helps drivers to retain control over the speed of their vehicle (Hellier et al., 2011). Our findings suggest that this may be particularly true for older adults, who appear to rely more heavily than younger adults upon the presence of congruent auditory input in order to govern their speed effectively and consistently. More recent initiatives have highlighted the need to be selective in terms of the frequencies that are attenuated, such that useful auditory information (e.g., engine rpm) is still transmitted to the driver, while repetitive and overrepresented sounds (e.g., road and tire noise at high speed) are reduced (e.g., Duan, 2011). Our findings indicate that this selective approach to active noise cancelation could be important for the safety of older drivers who may rely more heavily on auditory cues for accurate and reliable speed control.

In the current investigation we only considered the impact of congruent sensory cues and thus future research should endeavor to explore the outcomes associated with incongruent sensory cues. In our daily lives, we are immersed in sensory signals and good performance is contingent not only upon our capacity to combine related sensory cues but also upon our capacity to segregate unrelated cues (Meredith et al., 1987). In light of our observation that the performance facilitation associated with congruent cues (e.g., Laurienti et al., 2006; Peiffer et al., 2007) appears to generalize to more complex multisensory tasks, it stands to reason that the performance decrements associated with incongruent cues (e.g., Guerreiro et al., 2013; Setti et al., 2013) may also generalize to real-world, multisensory tasks. Future investigations will aim to characterize both the performance enhancements and the performance decrements associated with age differences in multisensory integration in order to fully appreciate the functional consequences that they may carry. Our research group is also exploring whether age differences in multisensory integration extend to other sensory cue combinations in the context of self-motion perception such as visual-vestibular cue integration (Ramkhalawansingh et al., 2015).

## Author Contributions

RR, JC, and BK conceived and designed the project. BH facilitated the design and implementation of the experiment by programming the driving simulation paradigm. RR and SS performed data acquisition and RR and JC performed data analysis and data interpretation. RC and JC drafted the manuscript and all authors made substantial, critical contributions to the revision and approval of the version to be published. All of the aforementioned authors agree to be accountable for the accuracy and integrity of the work.

## Conflict of Interest Statement

The authors declare that the research was conducted in the absence of any commercial or financial relationships that could be construed as a potential conflict of interest.
